# Finding flaws in the spatial distribution of health workforce and its influential factors: An empirical analysis based on Chinese provincial panel data, 2010–2019

**DOI:** 10.3389/fpubh.2022.953695

**Published:** 2022-12-14

**Authors:** Qian Bai, Xinyu Ke, Lieyu Huang, Liming Liu, Dongmei Xue, Ying Bian

**Affiliations:** ^1^State Key Laboratory of Quality Research in Chinese Medicine, Institute of Chinese Medical Sciences, University of Macau, Macau, China; ^2^Department of Public Health and Medicinal Administration, Faculty of Health Sciences, University of Macau, Macau, China; ^3^Office of Policy and Planning Research, Chinese Center for Disease Control and Prevention, Beijing, China; ^4^School of Traditional Chinese Medicine, Beijing University of Chinese Medicine, Beijing, China

**Keywords:** health workforce, spatial distribution, influential factor, spatial econometric model, China

## Abstract

**Background:**

The maldistributions of the health workforce showed great inconsistency when singly measured by population quantity or geographic area in China. Meanwhile, earlier studies mainly employed traditional econometric approaches to investigate determinants for the health workforce, which ignored spillover effects of influential factors on neighboring regions. Therefore, we aimed to analyze health workforce allocation in China from demographic and geographic perspectives simultaneously and then explore the spatial pattern and determinants for health workforce allocation taking account of the spillover effect.

**Methods:**

The health resource density index (HRDI) equals the geometric mean of health resources per 1,000 persons and per square kilometer. First, the HRDI of licensed physicians (HRDI_P) and registered nurses (HRDI_N) was calculated for descriptive analysis. Then, global and local Moran's I indices were employed to explore the spatial features and aggregation clusters of the health workforce. Finally, four types of independent variables were selected: supportive resources (bed density and government health expenditure), healthcare need (proportion of the elderly population), socioeconomic factors (urbanization rate and GDP per capita), and sociocultural factors (education expenditure per pupil and park green area per capita), and then the spatial panel econometric model was used to assess direct associations and intra-region spillover effects between independent variables and HRDI_P and HRDI_N.

**Results:**

Global Moran's I index of HRDI_P and HRDI_N increased from 0.2136 (*P* = 0.0070) to 0.2316 (*P* = 0.0050), and from 0.1645 (*P* = 0.0120) to 0.2022 (*P* = 0.0080), respectively. Local Moran's I suggested spatial aggregation clusters of HRDI_P and HRDI_N. For HRDI_P, bed density, government health expenditure, and GDP had significantly positive associations with local HRDI_P, while the proportion of the elderly population and education expenditure showed opposite spillover effects. More precisely, a 1% increase in the proportion of the elderly population would lead to a 0.4098% increase in HRDI_P of neighboring provinces, while a 1% increase in education expenditure leads to a 0.2688% decline in neighboring HRDI_P. For HRDI_N, the urbanization rate, bed density, and government health expenditure exerted significantly positive impacted local HRDI_N. In addition, the spillover effect was more evident in the urbanization rate, with a 1% increase in the urbanization rate relating to 0.9080% growth of HRDI_N of surrounding provinces. Negative spillover effects of education expenditure, government health expenditure, and elderly proportion were observed in neighboring HRDI_N.

**Conclusion:**

There were substantial spatial disparities in health workforce distribution in China; moreover, the health workforce showed positive spatial agglomeration with a strengthening tendency in the last decade. In addition, supportive resources, healthcare needs, and socioeconomic and sociocultural factors would affect the health labor configuration not only in a given province but also in its nearby provinces.

## Introduction

The health workforce is an essential element that assures the functioning of any health system (1), as well as its sustainable progress toward universal health coverage (2). The distribution of the health workforce affects the availability and accessibility of health service delivery to the public. Although its allocation is conventionally measured by the ratio of health workforce quantity to population size (3), geographical accessibility of the health workforce has also been found to facilitate health service efficiency as well as population health (4, 5). Therefore, it has been pointed out that an ideal allocation is portrayed as health workforce being equitably distributed and accessible by the population, regardless of geographical disparities (2, 6). In practice, demographic and geographic maldistribution of the health workforce is a long-standing and global crisis, particularly in China, which possesses the largest population and a vast territory (7–10).

Many studies have evaluated the allocation status of various types of health workforce in China. However, with far more focus on demographical allocation, only a few studies have addressed the geographical allocation issue (11). Notably, the existing literature reached a virtually unanimous conclusion that health workforce, including physicians, nurses, and pharmacists, was relatively balanced and distributed by population, while the maldistribution was much more severe when evaluated by geographic area (12–14). Given this inconsistency, the health resource density index (HRDI) was proposed to measure health resource distribution from the perspectives of both population quantity and geographical area (15, 16). Since then, it has been applied and validated as an effective instrument for health resource allocation, including health institutions, beds, health workers, and health financing, avoiding the bias caused by a single population or geographical factor (11, 17–21).

Regional difference is another problem of health workforce allocation in China. By means of the HRDI, several studies revealed that health personnel are intensively dispersed in the eastern region of China, as well as predominant variations across provinces (17, 19, 20). Recently, the growing development of empirical spatial statistics had an impact on approaches available in the domain of the health workforce allocation. Previous studies have measured the geographic aggregation features of health workforce allocation using spatial techniques; while the majority has focused on demographical allocation (22–26), only few studies have taken account of the geographical allocation (11, 27). Chen et al. found the HRDI of health personnel presented with positive spatial autocorrelation; in other words, provinces with more intensive health personnel clustered, and vice versa (11). Similarly, Lin JP constructed a comprehensive HRDI of health resource including institutions, beds, and personnel and then assured the spatial aggregation of health resource allocation based on Moran's I and hot spot analysis (27). Although spatial techniques have the advantage of capturing in-depth geographic features, it has been seldom applied to measure population- and geographic area-adjusted health workforce allocation.

Understanding the determinants for health workforce allocation is essential to cope with regional variations. The distribution of health manpower is shaped by a mix of macro-regional factors, such as GDP per capita, taxable income, elderly ratio, health resources, education level, and living environment (28–30). Although the influential factors have been broadly investigated, these studies were mostly conducted by means of traditional econometric models (31), which assume the determinants only exert impacts on the local area. However, certain behavior or outcome of a given area would be more or less affected by its neighboring areas (32). As a result, it is necessary to incorporate the spillover effect, which could be simply understood as the impacts of certain factors on surrounding regions, for more reliable statistical interpretations. Only a few scholars have surveyed the spillover effect of contextual factors on health resources, including the health workforce in China (33–38). For example, Zhu et al. proposed that growth in health service demands in a province could increase the doctor density in the surrounding units (37). Another study revealed a positive spillover effect of government health expenditure and medical graduates on the health technician density (38). However, these studies have only explored the spillover effect of various factors on the demographic distribution of the health workforce, which might be different if geographic distribution is considered.

The health workforce should be fairly distributed on the basis of population density and geographic area for better health outcomes. However, previous studies have merely calculated the health labor density by population, regardless of the geographic size. Spatial approaches are less frequently used in the health workforce allocation. Therefore, this study aims to evaluate health workforce allocation using the HRDI, explore the spatial pattern of health workforce allocation, and finally investigate the impact of external factors on health workforce allocation with an emphasis on the spillover effect using spatial econometric models in China from 2010 to 2019.

## Materials and methods

### Data sources and variable selection

#### Data sources

This research utilized the panel data of 31 provinces in China (except Hongkong, Macau, and Taiwan) from 2010 to 2019. All variables were retrieved from the Health Statistics Yearbook of China, China Statistical Yearbook, and Education Statistics Yearbook of China.

#### Health workforce allocation

In China, licensed physicians and registered nurses account for approximately two-thirds of the total number of health laborers and play dominant roles during the course of diagnosis, treatment, and rehabilitation in clinical settings (39, 40). Given the heterogeneity of each province, the HRDI as a measurement for health workforce allocation has the merit of avoiding bias by a single aspect of population and geographic size (17, 18). Therefore, the HDRI of licensed physicians (HRDI_P) and registered nurses (HRDI_N) was calculated for health workforce allocation in this study. The formula used to calculate the HRDI is as follows (16):


$$\begin{array}{l} HRDI\;=\;\sqrt{\left(\frac{n_{i}}{p_{i}}\right)\;*\;\left(\frac{n_{i}}{a_{i}}\right)} \end{array}$$

where *n*_*i*_ denotes the number of licensed physicians or registered nurses and *p*_*i*_ and *a*_*i*_ represent the population/thousand and geographical area/square kilometer of the province i, respectively.

#### Independent variables

In the health labor market, the health workforce can arbitrarily choose their practice locations out of various considerations, such as pecuniary benefit, career prospect, and amenity in specific areas. Therefore, macro-regional factors possibly impact the flow of the health workforce. Based on previous studies (26–29) and data accessibility, we selected four categories of influential variables for health workforce allocation (Table 1).

**Table 1:** Dependent and independent variables in this research.

**Variable type**	**Variable name**	**Measurement**	**Code**	**Expected effects**
Dependent variables	Human resource for health	Health resource of density index of physician	HRDI_P	Dependent variable
		Health resource of density index of nurse	HRDI_N	Dependent variable
Independent variables	Supportive resources	Bed density per thousand people	BD	+
		Government health expenditure per capita	GHE	+
	Healthcare need	Proportion of elderly population (aged over 65)	EP	+
	Socioeconomic factors	Urbanization rate	UR	+
		GDP per capita	GDP	+
	Sociocultural factors	Education expenditure per pupil	EE	+
		Park green area per capita	PGA	+

(1) Supportive resources

Clinical practice is not a highly individualized profession but heavily depends on a complex of supportive resources such as material and capital investments (41). Government health expenditure, as a crucial part of health institutions' budget, enables to raise the health technicians' income and upgrades facilities within institutions (42). Meanwhile, the quantity of beds is the necessary supportive facility for clinical practice and is closely associated with the scale and level of health institutions in China. Health technicians have more opportunities to promote their expertise in higher level institutions, which appeals to health labor in turn. Therefore, we choose the average government health expenditure and bed density as the proxies of supportive resources for a certain province and assume positive associations between supportive resources with health workforce allocation.

(2) Healthcare needs

The health workforce in a certain area is partially driven by the medical needs of residents (43). It is necessary to differentiate health needs from health demands. The former is a normative indicator, such as how many health technicians we ought to have (37, 38), and the latter refers to the extent to which people are willing or able to pay for medical services; however, this indicator is, in general, difficult to be measured directly (37, 38). Thus, we aimed to explore the impact of healthcare needs on labor allocation in this study. Previous studies have pointed out the close relevance between demographic situations with medical needs (41). Accordingly, we identify the proportion of the elderly population (older than 65 years) to reflect healthcare needs. The elderly population is susceptible to various diseases and require more health services as a result. Thus, we assume the elderly proportion has a positive impact on health workforce allocation.

(3) Socioeconomic factors

As reported, a higher proportion of health labor is concentrated in wealthier and urban areas (28, 44). Regional economy is, in general, positively associated with the average income of residents. Meanwhile, urban areas possess well-designed infrastructure, such as transportation, healthcare, and educational facilities, which are attractive for the health workforce. GDP per capita and urbanization rate are representative of socioeconomic factors and are expected to attract more health workforce.

(4) Sociocultural factors

The choice of work location also can be interpreted from the view of utility function, which reflects the relative attractiveness of a certain area (45). Apart from increasing salary, health professionals also seek to maximize the utility and life quality of the whole family from several aspects, such as the education quality of offspring, recreational services, and surrounding environment (46). In consequence, we choose education expenditure per pupil and park green area per capita on behalf of local education status and living environment. Then, we speculate that better sociocultural factor serves as an impetus to absorb more health workforce.

(5) Spillover effects

Spillover effects refer to the effects of within-region factors on neighboring regions (32). Although the spillover effect originates from economic activities, empirical studies have verified its existence in health resources, services, and outcomes (33–38). On the contrary, there is no strict constraint on the flow of health professionals across the provinces in China. Based on theoretical and practical implications, we presume that there exist spillover effects of supportive resources, healthcare needs, and socioeconomic and sociocultural factors on the health workforce allocation of adjacent provinces.

### Methods

#### Spatial autocorrelation test

Global and local Moran's I indices are frequently employed to detect and investigate the spatial correlation of variables. Global Moran's I index aims to estimate the spatial agglomeration and divergence distribution of observations from the entire research scope (47), while local Moran's I index further explores the spatial distribution between the local unit and its adjacent neighbors (48). The formula to calculate Moran's I index is as follows:


$$\begin{array}{lll} Global\;Moran^{{}^{\prime }}s\;I\; & = & \;\frac{n\sum_{i=1}^{n}\sum_{j=1}^{n}w_{ij}\left(y_{i}-\bar{y}\right)\left(y_{j}-\bar{y}\right)}{\sum_{i=1}^{n}\sum_{j=1}^{n}w_{ij}{\sum_{i=1}^{n}\left(y_{i}-\bar{y}\right)}^{2}}\\ Local\;Moran^{{}^{\prime }}s\;I\; & = & \;\frac{n\left(y_{i}\;-\;\bar{y}\right)}{{\left(y_{i}\;-\;\bar{y}\right)}^{2}}\overset{n}{\underset{j=1}{\sum }}w_{ij}\left(y_{j}-\bar{y}\right) \end{array}$$

where *y*_*i*_ and *y*_*j*_ denote the HRDI_P or HRDI_N of the province i and province j, $\overset{\bar{}}{y}$ is the average value, and *w*_*ij*_ represents the spatial weight matrix between units i and j. We construct the inverse distance of the centroid spatial matrix in order to comply with Tobler's first law of geography (49).

The value of global Moran's I index is in the range of [-1, 1]. Global Moran's I>0 indicates positive spatial agglomeration, I <0 denotes negative spatial correlation, and I = 0 represents random spatial distributions. There is no limitation for the value of local Moran's I index, but its explanations are similar to global Moran's I index. Local Moran's I>0 indicates a positive correlation between observation and its neighbors; in other words, a higher value is surrounded by higher values, or a lower value is surrounded by lower values; and local Moran's I <0 implies negative spatial dependence including higher–lower values or lower–higher values cluster. The significance of global and local Moran's I indices is tested by the *z*-value, and the formula to calculate z value is as follows (50):


$$\begin{array}{l} Z_{I}\;=\;\frac{I\;-\;E\left[I\right]}{\sqrt{V\left[I\right]}} \end{array}$$

where *E*[*I*] and *V*[*I*] are expectation and standard deviation, respectively, which are calculated by the following equations:


$$\begin{array}{lll} E\left[I\right]\; & = & \;\frac{-1}{\left(n-1\right)}\\ V\left[I\right]\; & = & \;E\left[I^{2}\right]-{E\left[I\right]}^{2} \end{array}$$

The analysis of global and local Moran's I indices is performed by GeoDa 1.20 software.

#### Spatial panel econometric model

There are three traditional spatial econometric models for panel data: the spatial panel lag model (SPLM), spatial panel error model (SPEM), and spatial panel Durbin model (SPDM).

The spatial econometric model takes account of spatial interactions between dependent and independent variables. To identify the optimal spatial model, we constructed and compared three commonly used spatial econometric models: SPLM, SPEM, and SPDM. These models have different assumptions regarding spatial interaction. In the SPLM, spatial interaction is believed to exist in dependent variables, which means that the HRDI_P or HRDI_N of a given province would be affected by the HRDI_P or HRDI_N of its adjacent provinces in this research; the SPEM confines spatial interaction within the error term, while the SPDM considers spatial effects of both dependent and independent variables (51). Accordingly, the formulas to calculate the SPLM, SPEM, and SPDM in this study are given as follows:

Spatial panel lag model (SPLM):


$$\begin{array}{lll} LnY_{it} & = & \rho WLnY_{it}+\beta_{1}LnBD_{it}+\beta_{2}LnGHE_{it}+\beta_{3}LnEP_{it}\\ & + & \beta_{4}LnUR_{it}+\beta_{5}LnGDP_{it}+\beta_{6}LnEE_{it}+\beta_{7}LnPGA_{it}\\ & + & \alpha +\mu_{i}+\gamma_{t}+\varepsilon_{it} \end{array}$$

Spatial panel error model (SPEM):


$$\begin{array}{lll} LnY_{it} & = & \beta_{1}LnBD_{it}+\beta_{2}LnGHE_{it}+\beta_{3}LnEP_{it}+\beta_{4}LnUR_{it}\\ & + & \beta_{5}LnGDP_{it}+\beta_{6}LnEE_{it}+\beta_{7}LnPGA_{it}+\alpha +\mu_{i}+\gamma_{t}\\ & + & \varepsilon_{it}\\ \varepsilon_{it} & = & \lambda W\varepsilon_{it}+\delta_{it} \end{array}$$

Spatial panel Durbin model (SPDM):


$$\begin{array}{lll} LnY_{it} & = & \rho WLnY_{it}+\beta_{1}LnBD_{it}+\beta_{2}LnGHE_{it}+\beta_{3}LnEP_{it}\\ & + & \beta_{4}LnUR_{it}+\beta_{5}LnGDP_{it}+\beta_{6}LnEE_{it}+\beta_{7}LnPGA_{it}\\ & + & \theta_{1}WLnBD_{it}+\theta_{2}WLnGHE_{it}+\theta_{3}WLnEP_{it}\\ & + & \theta_{4}WLnUR_{it}+\theta_{5}WLnGDP_{it}+\theta_{6}WLnEE_{it}\\ & + & \theta_{7}WLnPGA_{it}+\alpha +\mu_{i}+\gamma_{t}+\varepsilon_{it} \end{array}$$

where *Y*_*it*_ is the vector of the dependent variable HRDI_P or HRDI_N of the province i in year t; W represents the spatial weight matrix; γ_*t*_ and μ_*i*_ denote time and spatial fixed effect, respectively; ε_*it*_ is the random error term; α denotes the intercept of regression; ρ is the spatial autoregressive coefficient; λ is the spatial effects of random error; β_*i*_ (*i* = 1, 2…7) represents the influence of explanatory variables of the province i on explained variables of province i; and θ_*i*_ (*i* = 1, 2…7) describes the influence of explanatory variables of neighboring provinces on the province i.

To minimize the heteroscedasticity as much as possible, all variables were transformed into a logarithmic form (38). Previous research indicates that multicollinearity is not a concern in a regression model if the correlation coefficient is <0.85 (50). The correlations between all independent variables are displayed in Table 2, suggesting that multicollinearity was not an issue in this study.

**Table 2:** Pearson correlation analysis between independent variables.

	**LNBD**	**LNGHE**	**LNEP**	**LNUR**	**LNGDP**	**LNEE**	**LNPGA**
LNBD	1.0000						
LNGHE	0.5728^**^	1.0000					
LNEP	0.4654^**^	0.0195	1.0000				
LNUR	0.2806^**^	0.1925^**^	0.4886^**^	1.0000			
LNGDP	0.3958^**^	0.4863^**^	0.4236^**^	0.8545^**^	1.0000		
LNEE	0.4837^**^	0.7986^**^	0.2208^**^	0.6139^**^	0.8270^**^	1.0000	
LNPGA	0.3330^**^	0.3535^**^	0.3230^**^	0.8320^**^	0.8125^**^	0.6611^**^	1.0000

** *P* < 0.05.

BD: bed density per thousand people; GHE: government health expenditure per capita; EP: proportion of elderly population; UR: urbanization rate; EE: education expenditure per pupil; PGA: park green area per capita.

To ensure the robustness of the final regression model, the Lagrange multiplier (LM), likelihood ratio (LR), and Wald tests were conducted to determine the optimal model out of the SPLM, SPEM, and SPDM at first. Then, the Hausman test was performed to decide whether the fixed effect or random effect model was more appropriate. Finally, AIC and BIC were the major basis to decide the specified regression model. All the analyses were conducted in STATA 14.1. A *P* < 0.05 was considered statistically significant for all analyses.

## Results

### Basic characteristics

**Figure 1:**
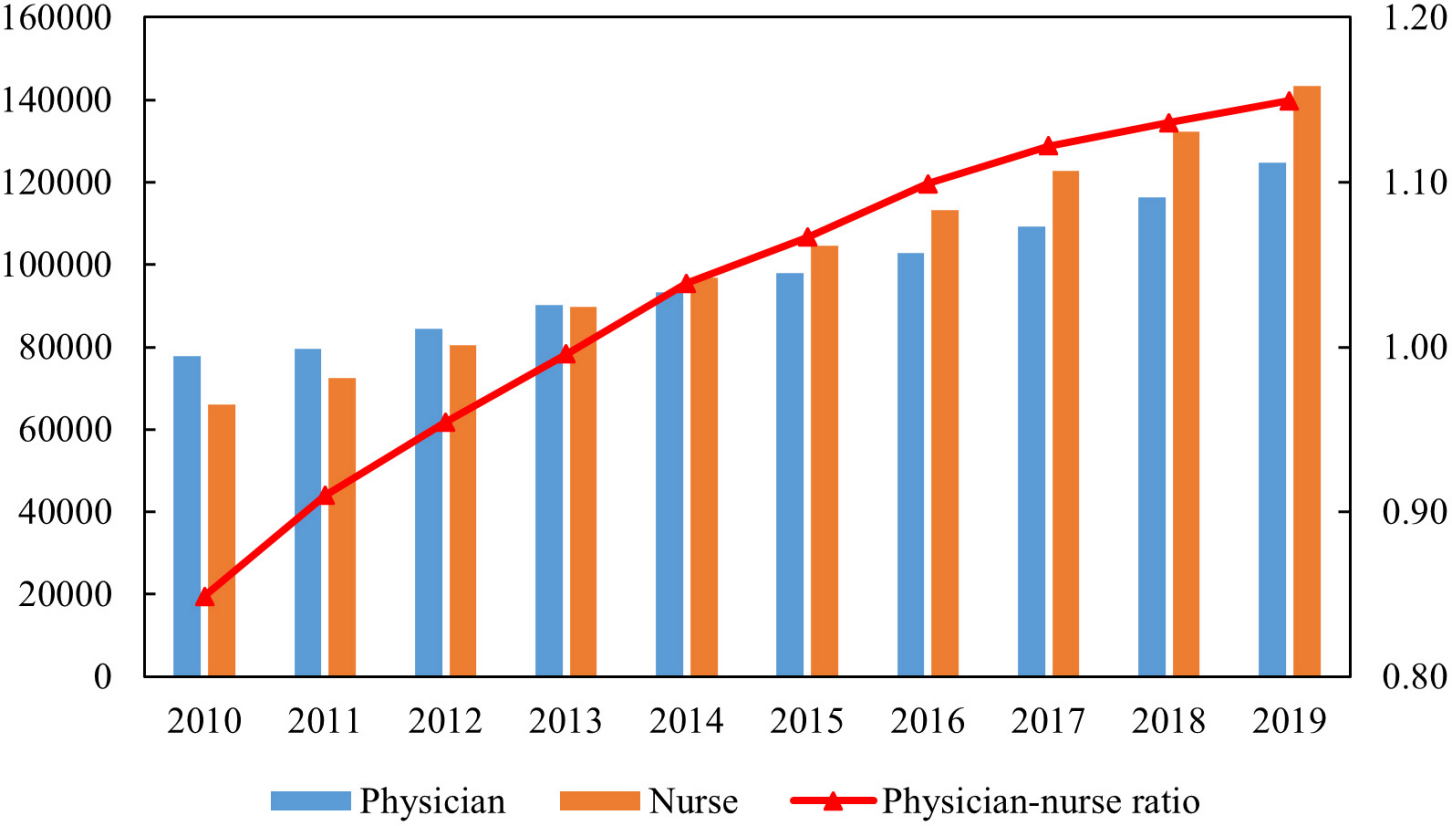
Physicians, nurses, and physician-to-nurse ratio from 2010 to 2019.

As shown in Figure 1, the number of physicians and nurses presented an increasing trend during the research period. Notably, the physician-to-nurse ratio changed from 1:0.85 in 2010 to 1:1.15 in 2019 in China. However, nearly one-third of its provinces were faced with an inverted physician-to-nurse ratio below 1:1, including Zhejiang, Tianjin, and Hebei in the eastern region; Gansu, Qinghai, Inner Mongolia, and Tibet in the west; and Heilongjiang, Shanxi, and Jilin in the central region on average (Supplementary Table S1).

### Spatial autocorrelation analysis

**Figure 2:**
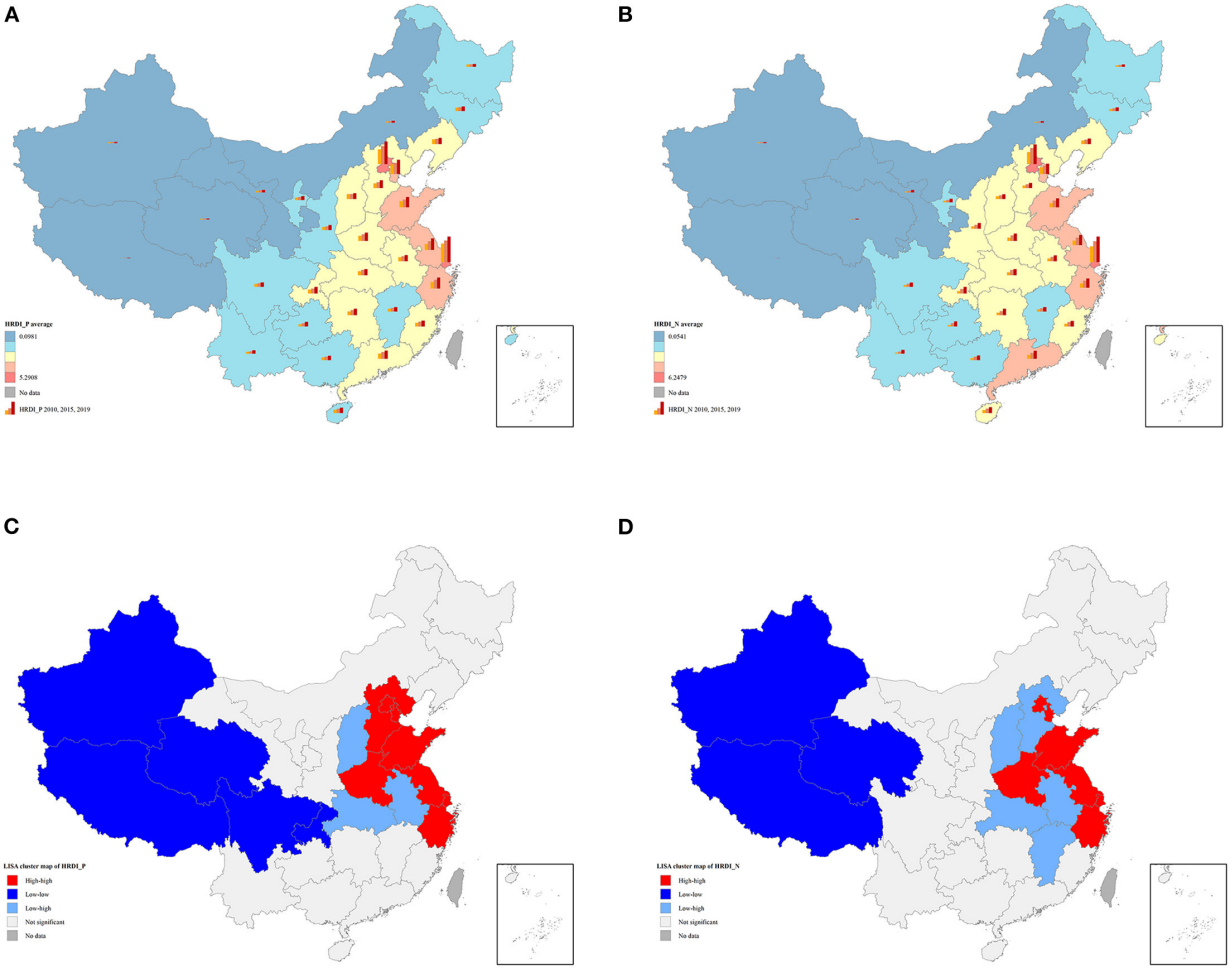
**(A)** Spatial distribution of HRDI_P; **(B)** spatial distribution of HRDI_N; **(C)** univariate local indicator of spatial association cluster map of HRDI_P in 2019; **(D)** univariate local indicator of spatial association cluster map of HRDI_N in 2019. The average value of HRDI_P and HRDI_N in **(A,B)** was divided into five levels based on natural breaks (Jenks). These figures are drawn based on the standard map from the Resource and Environment Science and Data Center (https://www.resdc.cn/Default.aspx).

Choropleth maps explicitly revealed similar geographical distribution in HRDI_P and HRDI_N in provinces with higher value of scattering in the eastern region, while those with a lower value were mainly located in the western region (Figures 2A,B). There was also empirical evidence of spatial autocorrelation of the two dependent variables. Global Moran's I index of HRDI_P and HRDI_N increased from 0.2236 to 0.2316 and from 0.1645 to 0.2022 in a decade, respectively, indicating gradually strengthened positive spatial autocorrelation (Table 3). Furthermore, we draw the cluster features of HRDI_P and HRDI_N in 2019 (Figures 2C,D). They were quite similar, except for Hebei, which belonged to the high–high cluster in HRDI_P but shifted to a low–high cluster in HRDI_N. In general, the high–high cluster area is mainly situated in the east region including Beijing, Tianjin, Shandong, Henan, Jiangsu, Zhejiang, Shanghai; the low–high cluster in the central region, such as Shanxi, Hubei, and Anhui; and the low–low cluster in the west represented by Xinjiang, Tibet, and Qinghai.

**Table 3:** Global Moran's I index of HRDI_P and HRDI_N.

**Year**	**HRDI_P**			**HRDI_N**		
	**Moran's I**	***Z*** **-value**	***P*** **-value**	**Moran's I**	***Z*** **-value**	***P*** **-value**
2010	0.2136	3.4370	0.0070^***^	0.1645	2.8881	0.0120^**^
2011	0.2080	3.3429	0.0100^**^	0.1678	2.9082	0.0120^**^
2012	0.2168	3.4425	0.0050^***^	0.1760	2.9981	0.0100^***^
2013	0.2238	3.5271	0.0050^***^	0.1853	3.1187	0.0090^***^
2014	0.2235	3.5272	0.0050^***^	0.1887	3.1530	0.0090^***^
2015	0.2255	3.5274	0.0050^***^	0.1909	3.1550	0.0090^***^
2016	0.2247	3.4989	0.0050^***^	0.1938	3.1761	0.0090^***^
2017	0.2275	3.5146	0.0050^***^	0.1951	3.1910	0.0090^***^
2018	0.2281	3.5225	0.0050^***^	0.2007	3.2520	0.0080^***^
2019	0.2316	3.5450	0.0050^***^	0.2022	3.2593	0.0080^***^

*** *P* < 0.01,

** *P* < 0.05.

### Spatial econometric regression

Table 4 shows the constraint statistics for sorting out the optimal model. Statistics for LM-error and LM lag tests were significant, indicating the spatial econometric model was more appropriate. Furthermore, LR error and Wald-error tests rejected its null hypothesis, indicating that the SPDM could not be simplified into the SPEM. Similarly, the SPDM could not be alternated by the SPLM based on LR lag and Wald lag estimations.

**Table 4:** Constraint statistics of the spatial econometric models.

**Test**	**LNHRDI_P**	**LNHRDI_N**
Moran's I	16.809^***^	16.529^***^
LM-error	248.210^***^	239.826^***^
Robust LM-error	188.455^***^	184.077^***^
LM-lag	59.804^***^	56.014^***^
Robust LM-lag	0.050	0.266
LR error	28.24^***^	56.59^***^
LR lag	20.50^***^	61.44^***^
Wald error	29.55^***^	62.82^***^
Wald lag	20.73^***^	69.93^***^

*** *P* < 0.01.

Regarding the specific type of the SPDM, the Hausman test was crucial to decide the fixed effect or random effect. Finally, the SPDM with a spatial random effect was chosen for HRDI_P (Hausman test = 19.51, *P* = 0.1916). However, the Hausman test of HRDI_N was significant (Hausman test = 30.39, *P* = 0.0106), a sign of the fixed effect model. Furthermore, the lowest AIC and BIC appeared in the SPDM with a two-way fixed effect for HRDI_N (Table 5), which was chosen as the appropriate model for HRDI_N.

**Table 5:** Spatial econometric models for HRDI_P and HRDI_N.

**Variables**	**LNHRDI_P (SDPM with spatial random effects)**	**LNHRDI_N (SDPM with spatial fixed effects)**	**LNHRDI_N (SDPM with time fixed effects)**	**LNHRDI_N (SDPM with two-way fixed effects)**
LNBD	0.2758^**^ (2.00)	0.4830^***^ (7.84)	0.1481 (0.74)	0.4987^***^ (9.20)
LNGHE	0.1024^*^ (1.70)	0.1508^***^ (3.50)	−0.3031^*^ (−1.94)	0.1055^***^ (2.70)
LNEP	−0.0352 (−0.78)	−0.0149 (−0.39)	0.7358^***^ (5.01)	−0.0498 (−1.42)
LNUR	0.3383^*^ (1.65)	0.8290^***^ (8.48)	2.1502^***^ (7.78)	0.8367^***^ (9.69)
LNGDP	0.1464^**^ (1.98)	0.0417 (0.96)	−0.3759^***^ (−2.71)	0.0586 (1.54)
LNEE	0.0175 (0.36)	0.0800^**^ (2.45)	0.3505^**^ (2.58)	−0.0468 (−1.38)
LNPGA	0.0334 (0.75)	0.0141 (0.47)	−0.1392 (−1.65)	−0.0212 (−0.79)
W^*^LNBD	−0.1955 (−0.97)	0.0609 (0.49)	−1.7514^***^ (−3.81)	0.3528^***^ (2.92)
W^*^LNGHE	−0.0716 (−0.95)	−0.0797 (−1.37)	−0.3515 (−0.91)	−0.2229^**^ (−2.24)
W^*^LNEP	0.2542^***^ (3.11)	0.2493^***^ (4.28)	−0.3931 (−0.97)	−0.2580^***^ (−2.90)
W^*^LNUR	−0.0684 (−0.24)	0.6665^***^ (2.83)	−0.0168 (−0.02)	1.6204^***^ (6.31)
W^*^LNGDP	0.0464 (0.52)	0.0375 (0.49)	2.3340^***^ (6.20)	0.0502 (0.62)
W^*^LNEE	−0.1559^**^ (−2.33)	−0.1421^***^ (−2.74)	−0.7955^**^ (−2.47)	−0.5167^***^ (−6.64)
W^*^LNPGA	0.0485 (1.10)	0.0785^*^ (1.86)	−1.6285^***^ (−6.71)	−0.0842^*^ (−1.86)
ρ	0.4538^***^ (4.48)	−0.0621 (-0.66)	0.3269^***^ (3.28)	−0.4678^***^ (−4.46)
AIC	−852.981	−1066.616	216.408	−1144.031
BIC	−785.723	−1006.831	276.1931	−1084.246
Log-Likelihood	44.4906	549.3082	−92.2040	588.0153
Adjusted *R*^2^	0.5910	0.5597	0.3901	0.4992
Obs	310	310	310	310

* *P* < 0.1,

** *P* < 0.05,

*** *P* < 0.01.

BD: bed density per thousand people; GHE: government health expenditure per capita; EP: proportion of elderly population; UR: urbanization rate; EE: education expenditure per pupil; PGA: park green area per capita.

It was notable that the estimated coefficients of the SPDM could not be directly interpreted as elasticities as OLS regression (51). The direct, indirect, and total effect of each independent variable on LNHRDI_P and LNHRDI_N are shown in Tables 6, 7.

**Table 6:** Direct, indirect, and total effects of independent variables on LNHRDI_P.

**Variables**	**Direct effects**	**Indirect effects**	**Total effects**	**Spillover effect**
LNBD	0.2734^*^ (1.92)	−0.1365 (−0.42)	0.1369 (0.37)	No
LNGHE	0.0981^*^ (1.73)	−0.0380 (−0.36)	0.0601 (0.59)	No
LNEP	−0.0119 (−0.29)	0.4098^***^ (3.23)	0.3979^***^ (3.20)	Yes
LNUR	0.3394 (1.57)	0.1903 (0.37)	0.5297 (0.86)	No
LNGDP	0.1603^**^ (2.18)	0.1977 (1.18)	0.3579^**^ (2.12)	No
LNEE	0.0048 (0.11)	−0.2688^**^ (−2.00)	−0.2640^*^ (−1.84)	Yes
LNPGA	0.0382 (0.83)	0.1150 (1.26)	0.1532 (1.34)	No

* *P* < 0.1,

** *P* < 0.05,

*** *P* < 0.01.

BD: bed density per thousand people; GHE: government health expenditure per capita; EP: proportion of elderly population; UR: urbanization rate; EE: education expenditure per pupil; PGA: park green area per capita.

**Table 7:** Direct, indirect, and total effects of independent variables on LNHRDI_N.

**Variables**	**Direct effects**	**Indirect effects**	**Total effects**	**Spillover effect**
LNBD	0.4942^***^ (8.29)	0.0834 (0.91)	0.5776^***^ (7.68)	No
LNGHE	0.1188^***^ (3.00)	−0.1946^**^ (−2.58)	−0.0758 (−1.09)	Yes
LNEP	−0.0321 (−0.88)	−0.1729^**^ (−2.55)	−0.2049^***^ (−3.32)	Yes
LNUR	0.7607^***^ (8.00)	0.9080^***^ (5.47)	1.6687^***^ (11.22)	Yes
LNGDP	0.0578 (1.48)	0.0171 (0.26)	0.0749 (1.38)	No
LNEE	−0.0154 (−0.46)	−0.3681^***^ (−6.49)	−0.3834^***^ (−5.71)	Yes
LNPGA	−0.0171 (−0.59)	−0.0520 (−1.52)	−0.0691^*^ (−1.87)	No

* *P* < 0.1,

** *P* < 0.05,

*** *P* < 0.01.

BD: bed density per thousand people; GHE: government health expenditure per capita; EP: proportion of elderly population; UR: urbanization rate; EE: education expenditure per pupil; PGA: park green area per capita.

Supportive resources, healthcare needs, and socioeconomic and sociocultural factors were found to affect HRDI_P in a different degree. First, bed density, GDP, and government health expenditure would significantly positively influence local HRDI_P, manifesting as a 1% increase in bed density, GDP, and government health expenditure, leading to 0.27%, 0.16%, and 0.10% growth in HRDI_P of the given province, respectively. From the view of spillover effects, a 1% increase in the proportion of the elderly population and education expenditure per pupil brought about a 0.41% increase and a 0.27% decline in HRDI_P for neighboring provinces, respectively (Table 6).

As for HRDI_N, the urbanization rate, bed density, and government health expenditure exerted significantly positive impacts on local HRDI_N, a 1% growth of which was equal to 0.76%, 0.49%, and 0.12% increase in HRDI_N of the province itself, respectively. Evident spillover effects for HRDI_N were observed in the urbanization rate, GDP, government health expenditure, and proportion of the elderly population. Among these factors, the urbanization rate had a substantial and positive spillover effect as a 1% increase in the local urbanization rate led to a 0.91% increase of HRDI_N in all the neighboring provinces. On the contrary, the spillover effects of education expenditure, government health expenditure, and proportion of the elderly population were obviously negative on the HRDI_N of neighboring provinces (Table 7).

## Discussion

Our study aims to identify the existing flaws in the spatial distribution of health workforce and investigate determinants for the health workforce, which might be beneficial to future remedies concerning this issue. The results revealed substantial spatial disparities in the health workforce in China, as well as an increasing positive spatial aggregation in the health workforce. In addition, macro-regional factors including supportive resources, healthcare needs, and socioeconomic and sociocultural factors would shape the workforce allocation of not only the province but also of nearby areas.

Regardless of the sustainable growth in the health workforce, the problem of unreasonable structure between physicians and nurses deserves research attention, which has also been pointed out by previous studies (25, 52). The ratio of doctors to nurses was estimated at 1:1.5 in 2019 in China, which was far from the value of 1:3.16 in Korea and 1:4.62 in the United States in the same year based on OECD data. In general, nurses have relatively lower prestige than physicians but still bear a heavy workload, which might be the reason for the long-standing severe shortage of nursing staff (28, 53). In response to the escalating medical burden, the government should continue to make tremendous efforts in regulating health labor configuration, especially the structure of physicians and nurses.

Our findings also revealed obvious spatial disparities in physicians and nurses allocation. The HRDI of physicians and nurses was the highest in the eastern region, while the lowest value was found in three western provinces, namely, Tibet, Qinghai, and Xinjiang. However, prior studies have evaluated the demographic allocation of health labor and revealed that Tibet and Xinjiang obtained sufficient health labor stock (12, 23, 24). The inconsistency is related to the population-oriented allocation policy of health labor in China. When taking account of the geographic area, large, sparsely populated regions like Tibet would possess less health workforce. Indeed, the longer travel distance might delay health service utilization, followed by poor health outcomes (54). Therefore, health labor should be scientifically distributed on the basis of population size and geographic area. According to “Health China 2030,” it has been recommended to assign health labor, taking both population size and territory into account, to improve the geographic accessibility of health services. Moreover, our results also pointed out that the spatial aggregation of physicians and nurses even aggravated in the last decade, as evidenced by a slightly increase of global Moran's I index in HRDI_P and HRDI_N in our study. Similarly, Zhang et al. found global Moran's I index of nurse density increased from 0.113 in 1998 to 0.198 in 2018 (25), and Chen et al. observed the HRDI of health personnel increased from 0.055 in 2009 to 0.103 in 2018 through global Moran's I analysis (11). These figures imply the enlarging spatial heterogeneity in health labor allocation. Specifically, high–high clusters for physicians and nurses are mainly located in the eastern region, while low–low clusters are dispersed in the western region. The flexible mobility of health manpower might account for this spatial heterogeneity. From one perspective, the regional medical resources including institutions and equipment determine the potential capacity of health labor in a certain region. Pan et al. pointed out that western provinces were at a decided disadvantage in terms of bed density (55), and Shi et al. revealed that 46.68% of elite health institutions were concentrated in the eastern coastal areas (56). As a result, provinces in the eastern region are able to attract and retain a larger quantity of health professionals with the advantage of abundant medical resources. In addition, provinces in the eastern region are, in general, economically developed; thus, health professionals might have more incentives to work in these areas driven by a higher salary. The marketization possibly further facilitates the outflow of health labor from underdeveloped to developed regions and then accelerate spatial agglomeration (57). In this case, it is necessary for the government to carry out more effective regional-specific policies targeted at backward areas with respect to health workforce configuration based on in-depth understanding of its influential factors.

Empirically, health workforce allocation was significantly associated with local macro-regional status, including supportive resources and socioeconomic factors. As one major medical resource, bed density was positively related to the local HRDI_P and HRDI_N in our study, which is consistent with prior research (31). It was also noted that the impact of bed density on HRDI_N was nearly two times higher than that on HRDI_P in this study. The possible explanation might be that some subtypes of physicians mainly provided outpatient services, such as TCM doctors and dentists (37), while nurses were more closely connected with inpatient services (38, 58). Therefore, nurse allocation was more sensitive to bed density. Regarding capital investment, government health expenditure exerted significant positive impacts on HRDI_P and HRDI_N, with coefficients of 0.0981 and 0.1188, respectively. This view has been broadly confirmed by prior studies (36, 37, 57). A national survey in China also showed an enlarging government subsidy contributed to reduce the turnover rate among health technicians (59). Hence, abundant health resources are the driving forces for attracting health labor. Among socioeconomic factors, GDP and urbanization rate were positively associated with HRDI_P and HRDI_N. Yu et al. found that GDP and urbanization rate would positively affect the configure of medical resources, with coefficient values of 0.0389 and 1.4898, respectively (60). Apart from a decent salary, favorable socioeconomic status stimulates residents' health demands and utilization (61), which encourage hiring more health laborers in the local health system.

Remarkably, regional characteristics, especially healthcare needs and sociocultural factors, exerted spillover effects on the health workforce allocation in nearby provinces. The elderly proportion was associated with health labor in surrounding areas but in an opposite manner. A 1% increase in the elderly proportion was linked to a 0.4098% increase in HRDI_P but a 0.1729% reduction in HRDI_N in neighboring areas. Owing to the convenient transportation, residents frequently seek cross-regional medical services (33, 62). In general, the elderly with chronic or mobility-impaired illnesses receive long-term treatment together with overwhelming nursing demands in local health institutions (63, 64), which possibly induce hiring of nurses from neighboring regions. In addition, negative spillover effects of education expenditure were observed on the HRDI_P and HRDI_N of neighboring regions. Several studies have already pointed out that health laborers had more probability to choose practice locations with a higher education level for the sake of their children (28, 30). In addition, a strong spillover effect was found in the urbanization rate, manifesting as a 1% increase in the urbanization rate was related to 0.9080% growth of HRDI_N in nearby areas. Similarly, previous studies have also identified a positive spillover effect of the urbanization rate on health professionals (33, 35, 65). The ongoing urbanization process facilitates to break through the barriers brought about by the administrative division of provinces (57), which accelerates the spillover effect on health labor allocation across regions.

This study also has some limitations. First, we only evaluated the spatial pattern and determinants of licensed physicians and registered nurses, but other types of health workforce may present with different features, which deserve further exploration. Second, it has been reported that individual factors such as gender, age, and education possibly affect workforce allocation, to some extent (28). Therefore, further investigation on the impact of individual factors might be beneficial to obtaining more comprehensive insights health workforce allocation.

## Conclusion

In summary, our study identified substantial spatial disparities in health workforce allocation in China. Moreover, the positive spatial aggregation in the health workforce has strengthened during the research period, a sign of expanding spatial heterogeneity. In addition, supportive resource, healthcare needs, and socioeconomic and sociocultural factors were found to be associated with health labor configuration not only in the given province but also in nearby provinces. Although the health workforce remains sustainable growth in quantity, it requires more intensive attentions whether the health workforce stock distributing within a country or region in a balanced manner.

## Data availability statement

The original contributions presented in the study are included in the article/Supplementary material, further inquiries can be directed to the corresponding author.

## Author contributions

YB and QB designed the study. QB conducted data analysis and drafted the manuscript. XK contributed to data collection and drafted the manuscript. LH, LL, and DX revised the manuscript. All authors have read and agreed to the published version of the manuscript.
