# Supplementary material for: Finding flaws in the spatial distribution of health workforce and its influential factors: An empirical analysis based on Chinese provincial panel data, 2010–2019
Source: Front Public Health. 2022 Dec 14;10:953695. doi: 10.3389/fpubh.2022.953695 (PMC9794860; doi:10.3389/fpubh.2022.953695)
Supplement: Supplementary file 1 [file Table_1.DOCX]

Table s1. Physician-nurse ratio of 31 provinces in China, 2010-2019

| Region | Province | Physician-nurse ratio |
| --- | --- | --- |
| Eastern region | Beijing | 1:1.08 |
|  | Tianjin | 1:0.91 |
|  | Hebei | 1:0.77 |
|  | Liaoning | 1:1.04 |
|  | Shanghai | 1:1.18 |
|  | Jiangsu | 1:1.06 |
|  | Zhejiang | 1:0.99 |
|  | Fujian | 1:1.13 |
|  | Shandong | 1:1.04 |
|  | Guangdong | 1:1.11 |
|  | Hainan | 1:1.29 |
| Western region | Inner Mongolia | 1:0.91 |
|  | Guangxi | 1:1.21 |
|  | Chongqing | 1:1.10 |
|  | Sichuan | 1:1.02 |
|  | Guizhou | 1:1.18 |
|  | Yunnan | 1:1.15 |
|  | Tibet | 1:0.55 |
|  | Shaanxi | 1:1.26 |
|  | Gansu | 1:0.98 |
|  | Qinghai | 1:0.97 |
|  | Ningxia | 1:1.06 |
|  | Xinjiang | 1:1.08 |
| Central region | Shanxi | 1:0.91 |
|  | Jilin | 1:0.90 |
|  | Heilongjiang | 1:0.95 |
|  | Anhui | 1:1.09 |
|  | Jiangxi | 1:1.15 |
|  | Henan | 1:1.02 |
|  | Hubei | 1:1.16 |
|  | Hunan | 1:1.01 |
| Overall | — | 1:1.05 |
